# Management and Prevention of Anaphylaxis

**DOI:** 10.12688/f1000research.7181.1

**Published:** 2015-12-22

**Authors:** Anne-Marie Irani, Elias G. Akl

**Affiliations:** 1 Department of Pediatrics, Virginia Commonwealth University, Richmon, Virginia, 23298, USA

**Keywords:** Anaphylaxis, management, prevention, epinephrine, hypersensitivity, food, exercise, asthma

## Abstract

Anaphylaxis prevalence has increased within the last few years. This may be due to a marked increase in allergic sensitization to foods especially in the pediatric population, as well as to an increase in outdoor recreational habits and the availability of new biologic medications.  Furthermore, guidelines for the diagnosis of anaphylaxis have been published, thus facilitating the recognition of this disorder. Diagnosis of anaphylaxis is mainly based on history and clinical criteria of organ system involvement. The serum tryptase assay is now commercially available and may be a helpful diagnostic tool in certain clinical situations involving hypotension, but not in the context of food-induced anaphylaxis. Treatment of anaphylaxis mainly involves the use of epinephrine as a first line medication for severe manifestations followed by  symptomatic management of specific  symptoms, such as antihistamines for urticaria and albuterol for wheezing. Although commonly practiced, treatment with systemic corticosteroids  is not supported by evidence-based literature. Observation in a medical facility for 4-6 hours is recommended to monitor for late phase reactions, although these rarely occur. Education is an essential component of management of a patient with a previous history of anaphylaxis, emphasizing early use of epinephrine and providing a written action plan. Referral to a board-certified allergist/immunologist is recommended to determine  the cause of the anaphylaxis as well as to rule out other potential conditions. In this review, our main focus will be on the treatment and prevention of anaphylaxis while providing our readers with a brief introduction to the diagnosis of anaphylaxis, its prevalence and its most common causes.

## Introduction: Definition, Prevalence, and Common Triggers

There are several accepted definitions of the term anaphylaxis in the medical literature, all of which share the common characteristic of a severe, life-threatening, generalized hypersensitivity reaction ^1– 3^. The term anaphylactoid reaction is no longer used based on the World Allergy Organization’s recommendation to further define anaphylaxis into immunologic, immunoglobulin E (IgE) mediated, and non-immunologic reactions ^2^. The lifetime prevalence of anaphylaxis is estimated at 1.6–5% based on a recent national telephone survey ^4, 5^. Food remains the most common overall outpatient cause of anaphylaxis across all ages combined ^6^, accounting for 30% of cases of fatal anaphylaxis ^6^. The most common foods that trigger anaphylaxis include peanuts, tree nuts, fish, and shellfish, with the addition of cow’s milk in children ^7, 8^. There has been a recent increase in patients with sesame seed anaphylaxis ^6^. Among medications, antibiotics and primarily penicillin are most commonly involved in subjects aged 18 years and above ^5^ followed by non-steroidal anti-inflammatory drugs (NSAIDs) ^9^. Other drugs implicated include biologics and monoclonal antibodies ^10, 11^. Anaphylaxis to insect stings has occurred in 3% of adults and 1% of children who have been stung ^12, 13^. Other less common causes of anaphylaxis include seminal fluid and vaccines or vaccine components ^6^. Exercise-induced anaphylaxis (EIAn) usually occurs with a co-trigger ^14– 16^ such as food ingestion. Patients with systemic mastocytosis are at increased risk of anaphylaxis from all causes, given the increase in mast cell burden. Anaphylaxis secondary to latex remains a concern in patients with spina bifida as well as in healthcare workers, but this has become less common, especially with hospitals using latex-free products throughout the country ^6^. Patients on allergen immunotherapy (AIT) also carry a small risk of 0.25%–1.3% of anaphylaxis ^6, 17, 18^, especially in the context of poorly controlled asthma ^6^. The risk of a fatal reaction from AIT is estimated at around 1 in 2.5 million injections ^6, 19, 20^. Idiopathic anaphylaxis remains a diagnosis of exclusion when extensive trigger identification fails.

## Diagnosis

History: the diagnosis of anaphylaxis relies principally on the history, including the time course of the event, such as history of exposure to a particular trigger, the time course between exposure and development of symptoms, and the evolution of symptoms and signs over minutes to hours.

Diagnostic criteria: the diagnostic criteria set forth by the National Institutes of Health (NIH) in 2006 were based on three clinical scenarios:

First, in the absence of an allergen, anaphylaxis is diagnosed by a rapid onset (minutes to hours) of a reaction that involves the skin, mucosal tissue, or both, alongside at least one of the following symptoms: respiratory compromise, reduced blood pressure, or symptoms of end organ dysfunction.

Second, after a likely allergen exposure, two or more of the following occur: involvement of the skin or mucosal tissue, respiratory symptoms, decreased blood pressure, and/or gastrointestinal involvement.

Third, in the case of a known allergen, reduced blood pressure alone is sufficient for the diagnosis of anaphylaxis ^1^.

Laboratory test: elevated serum tryptase levels can be detected within 15 minutes and up to 3 hours after the anaphylactic episode ^21– 23^. Levels greater than 11.5 ng/mL are considered elevated. Serum tryptase levels are rarely increased in the absence of shock or when food is the trigger ^6^. Baseline elevations of serum tryptase levels should prompt consideration of the diagnosis of systemic mastocytosis ^24^. A recent consensus document defined a significant acute elevation of serum tryptase to be equal to or greater than 1.2 times the baseline +2 ng/mL, indicating likely mast cell activation ^25^.

## Management

The management of anaphylaxis includes treatment of acute episodes and preventive measures including management of comorbidities, identification and avoidance of specific triggers, and select instances of immunomodulation.

### Acute treatment

The recommendations for acute treatment of anaphylaxis are largely based on expert opinion and consensus, as there are no randomized controlled studies for any of the pharmacologic therapies used.

All published guidelines clearly identify epinephrine as the first-line medication for the treatment of anaphylaxis ^1, 6^. Epinephrine 1:1000 (1 mg/mL) at a dose of 0.2–0.5 mg in adults and 0.01 mg/kg in children up to a maximum of 0.3 mg dosage should be used ^6^. Injection in a large muscle, usually the lateral thigh, results in better absorption of the medication ^26^. There are currently two commercially available doses of epinephrine autoinjectors in the United States: 0.15 mg (ideal for a 15 kg body weight) and 0.3 mg (ideal for a 30 kg body weight). In Europe, a third dose of 0.5 mg has been marketed but is not available for use in the US. It is common practice to prescribe the 0.15 mg dose to children weighing as low as 10 kg and the 0.3 mg dose to children after they reach a body weight of 24 kg. The practice parameters allow physicians to use epinephrine every 5–10 minutes and even at shorter intervals if deemed necessary ^6^. It is important to remember that patients on oral or even ophthalmic beta-blockers might not adequately respond to epinephrine ^27, 28^. In these patients, isotonic saline and intravenous glucagon given at a dose of 1–5 mg in adults and 20–30 µg/kg in children, up to a maximum of 1 mg, should be given, followed by an infusion at a rate of 5–15 µg/minute titrated to clinical response ^29– 31^. Depending on the setting (healthcare versus at home), intravenous fluids should be initiated to maintain adequate circulation ^32, 33^.

Another important consideration, which is often ignored, is to position the patient in the Trendelenburg position (lying flat on the back with legs elevated) in order to allow blood flow to the heart and to prevent the "empty ventricle syndrome" described by Pumphrey ^34^.

Other supportive measures could be considered as second-line therapy. These include oxygen use, H1 and H2 antihistamines for the treatment of hives, and albuterol for the treatment of bronchospasm. We recommend using a non-sedating antihistamine as opposed to the common practice of prescribing diphenhydramine, as the sedative effect might obscure possible central nervous system symptoms. Corticosteroids are not useful for the acute treatment of anaphylaxis but may be effective in preventing biphasic or protracted anaphylaxis. As a result, many centers will administer a single dose of systemic corticosteroids (orally or intravenously) after the patient has been stabilized ^35, 36^. A prolonged 3 or 5 day course is not indicated. The frequency of occurrence of biphasic reaction has been reported to be from as low as 1% to as high as 23% ^6, 37, 38^. These different estimates are likely due to varying definitions of anaphylaxis and the criteria used to identify a biphasic reaction. Using the NIH definitions for anaphylaxis in a retrospective chart review of two urban academic hospitals in Canada, Grunau *et al.* reported the incidence of a biphasic clinically important reaction to be 0.18% ^39^. Currently, expert consensus recommends observation in the emergency room for a period of at least 6 hours after stabilization ^6, 38, 40– 43^. Patients should be discharged home with a prescription for an epinephrine autoinjector (EpiPen), along with instructions for self-administration and a referral to an allergy/immunology specialist for diagnosis and prevention.

### Prevention

Long-term preventive measures include the recognition and management of risk factors for anaphylaxis in general, as well as measures directed to the specific triggers in particular.

It is important to identify and manage comorbid conditions that increase the risk of a severe anaphylactic reaction when poorly controlled. These include asthma, cardiovascular disease, and mastocytosis or mast cell activation syndrome. Furthermore, administration of certain medications such as beta-blockers may interfere with the therapeutic response to epinephrine as previously mentioned. Young children may not be able to recognize and report early symptoms of anaphylaxis, leading to a delay in administration of epinephrine. Adolescents and young adults often display risky behavior with regards to food avoidance and poor compliance in carrying the epinephrine autoinjector.

The next section will review preventive measures specific to the various diagnostic categories of anaphylaxis.

***Food-induced anaphylaxis*.** Avoidance of the confirmed food trigger requires lifelong vigilance, including education on reading food labels, informing family and friends, and caution while eating in public establishments. Given the difficulty in implementing complete food avoidance and the resultant negative effect on quality of life, clear and consistent information should be provided regarding the specific food triggers. In some patients, food challenges performed in a clinical setting may be necessary to assess the clinical significance of positive skin tests or serum IgE levels. Various forms of immunotherapy for food desensitization are currently being investigated, including oral, sublingual, and patch application ^44– 51^. Primary prevention of peanut allergy in high-risk infants with severe eczema and/or egg allergy was recently reported in a landmark study where early introduction of peanut between the ages of 4 and 11 months in infants with negative oral peanut challenge resulted in a rate of peanut allergy of 3% at 5 years of age compared to 17% in the group of infants who practiced peanut avoidance, an 86% relative risk reduction in infants with negative peanut skin tests ^52^.

***Medication-induced anaphylaxis*.** As with foods, an accurate determination of the offending medication is needed. In situations when the patient is receiving multiple medications simultaneously a detailed history is crucial. Standardized skin testing is available for penicillin only ^53^, although many protocols have been reported for other antibiotics and miscellaneous drugs ^54, 55^. Once identified, the offending medication should be avoided and alternative therapies used. In that respect, it is important to identify medications with potential cross-reactivity to the offending agent. If no alternative medication is adequate to treat the underlying condition, careful desensitization by administering incremental doses of the offending drug can be performed, often in an intensive care unit setting ^56, 57^. This procedure does not confer long-term tolerance to the drug, so future administration of the drug would once again require a desensitization procedure. Omalizumab, a monoclonal antibody against IgE, given subcutaneously for the treatment of difficult-to-control asthma as well as chronic spontaneous urticaria carries a 1 in 1000 risk of anaphylaxis, especially after the first three doses. Current expert opinion recommends injections to be administered at a medical facility, together with monitoring for 2 hours following the first three injections and for 30 minutes after subsequent injections. Patients are also advised to carry an epinephrine autoinjector for a period of 24 hours following the injections ^11^.

***Insect sting anaphylaxis*.** Anaphylaxis to insects occurs in 3% of adults and 0.4–0.8% of children who are stung ^6, 58^. History, as always, is key in identifying the insect, correlating the onset of symptoms to the sting event and helping in avoidance of future stings. Different insects build nests in different places: hornets build large nests in trees, yellow jackets in the ground, and wasps under houses or barns. Honeybees usually leave a stinger and build nests in tree hollows. Wasps, yellow jackets, and hornets are scavengers and are likely to be encountered in picnic areas where food is available. Fire ants build their nests in soil and often sting in a circular pattern multiple times. Patients with a history of insect sting hypersensitivity should be educated on avoidance of stings, carry an epinephrine autoinjector, and obtain a consultation with an allergist/immunologist in order to undergo specific serum IgE testing and skin testing to identify the culprit insects. Randomized controlled trials have demonstrated the development of long-lasting protection against anaphylaxis in most patients who are treated with subcutaneous venom immunotherapy for a period of 3–5 years ^6, 58– 61^. Venom extracts are available for honeybee, yellow jacket, white-faced hornet, yellow hornet, and wasp, and whole body extract is available for fire ant. Patients with mastocytosis and mast cell activation syndrome have an increased risk of anaphylaxis with insect sting, whereby the anaphylactic episode could be the presenting sign of the disorder ^62, 63^.

***Exercise-induced anaphylaxis*.** EIAn, as the name implies, is anaphylaxis induced by physical activity. The mechanism behind it is still not entirely clear. Symptoms usually start within a few minutes after exercise and include fatigue, flushing, itching, and urticaria. If exercise continues, symptoms may progress in severity with angioedema of the airways and death ^64, 65^. Often, a co-trigger is required for symptoms to develop, such as a specific (or any solid) food, NSAIDs, menstruation, alcohol, or even pollen exposure in sensitized individuals ^14, 66^. The risk of anaphylaxis with exercise may occur within 4–6 hours of food and alcohol ingestion and within 24 hours after administration of NSAIDs. The most common food trigger in the USA is wheat, followed by grains and seafood ^14, 15^. Potential co-triggers could be identified through skin testing and exercise challenge testing despite its low sensitivity. It is imperative to identify the co-triggers in order to provide education on avoidance. H2 antagonists should be avoided, as preliminary data show that they might interfere with the normal digestion of food and potentially lead to a more severe reaction ^16, 67^. Therefore, prevention is individualized to the patient and to the co-triggers. These patients can exercise regularly once the co-trigger is avoided for a period of time prior to exercise. They should be counseled to exercise with a partner at all times and should carry epinephrine for autoinjection. If early signs or symptoms develop, the patient should stop exercising in order to avoid progression ^6^.

***Allergy to galactose-alpha-1,3-galactose, also known as “alpha-gal”*.** More recently, a new cause of anaphylaxis has been linked to red meat consumption with a delayed onset of 3–5 hours or more after ingestion ^68, 69^. Patients usually report a history of a lone star tick bite 1–3 months prior to anaphylaxis ^70^. The pathogenesis is due to the development of an IgE response to a mammalian oligosaccharide epitope, galactose-alpha-1,3-galactose, known as alpha-gal, present in the tick and conserved in mammalian meat ^70^. A typical presentation would be a patient waking up in the middle of the night and collapsing on the way to the bathroom after ingestion of mammalian products for dinner. Episodes are sporadic ^70^. There is a commercially available serum test to detect IgE against alpha-gal. Avoidance of mammalian meat is recommended as well as availability of an epinephrine autoinjector.

***Idiopathic anaphylaxis*.** Idiopathic anaphylaxis remains a diagnosis of exclusion after extensive history and testing to rule out specific triggers, including foods, exercise, medications, and insect hypersensitivity. Laboratory workup to look for evidence of mast cell activation is indicated. Serum tryptase levels obtained at baseline, as well as within 3–4 hours of an acute episode, can be helpful in demonstrating acute mast cell activation ^23– 25^. Other mast cell mediator measurements of urinary metabolites include N-methylhistamine, leukotriene E4, and prostaglandin F2 alpha (PGF2alpha). An elevated basal serum tryptase level suggests the diagnosis of systemic mastocytosis. A bone marrow biopsy can also be considered ^70, 71^. In patients with elevated serum PGD2 (or its urinary metabolite, PGF2alpha), treatment with 650 mg aspirin twice a day is recommended ^72, 73^. Treatment with high-dose prednisone, 60–100 mg daily for 1–2 weeks along with non-sedating H1 and H2 antihistamines, followed by tapering the dose of prednisone on alternate days over a period of 3 months has also been shown to decrease the frequency and severity of anaphylactic episodes, but results in high toxicity ^74, 75^. Multiple reports ^76– 78^, as well as our own experience, have shown that treatment with omalizumab, a monoclonal anti-IgE antibody, can lead to decreased frequency of episodes and is very well tolerated. An epinephrine autoinjector should be carried at all times.

## Conclusions

Anaphylaxis is a potentially life-threatening condition. Given its high prevalence, 2–5% of the population, physicians of all specialties are likely to be tasked with the recognition and management of anaphylactic episodes. In this regard, several consensus guidelines, including the American, European and World Allergy Organization guidelines, have been published to facilitate this task ^1– 3^. A careful history and specialized testing to identify potential triggers are paramount in preventing future events. Measurements of mast cell mediators in biologic fluids can improve the diagnostic accuracy of anaphylaxis. Epinephrine remains the mainstay of treatment for acute episodes. Emerging therapies include the use of omalizumab as well as allergen-specific immunotherapy.
